# Ego- and Allocentric Visuospatial Neglect: Dissociations, Prevalence, and Laterality in Acute Stroke

**DOI:** 10.1037/neu0000527

**Published:** 2019-03-21

**Authors:** Nele Demeyere, Celine R. Gillebert

**Affiliations:** 1 Department of Experimental Psychology, University of Oxford; 2 Department of Experimental Psychology, University of Oxford, and Department of Brain and Cognition, University of Leuven

**Keywords:** spatial neglect, egocentric, object-based, stroke, lateralization

## Abstract

***Objective:*** Visuospatial neglect, whereby patients are unable to attend to stimuli on their contralesional side, is a neuropsychological condition commonly experienced after stroke. We aimed to investigate whether egocentric and allocentric neglect are functionally dissociable and differ in prevalence and laterality in the early poststroke period. ***Method:*** A consecutive sample of 366 acute stroke patients completed the Broken Hearts test from the Oxford Cognitive Screen. We evaluated the association between egocentric and allocentric neglect and contrasted the prevalence and severity of left-sided versus right-sided neglect. ***Results:*** Clinically, we found a double dissociation between ego- and allocentric neglect, with 50% of the neglect patients showing only egocentric neglect and 25% only allocentric neglect. Left-sided egocentric neglect was more prevalent and more severe than was right-sided egocentric neglect, though right-sided neglect was still highly prevalent in the acute stroke sample (35%). Left-sided allocentric neglect was more severe but not more prevalent than was right-sided allocentric neglect. At 6 months, in a representative subsample of 160 patients, we found neglect recovery rates to be 81% and 74% for egocentric and allocentric neglect, respectively. ***Conclusion:*** Dissociable ego- and allocentric neglect symptoms support a heterogeneous account of visuospatial neglect, which was shown to be highly prevalent for both the left and the right hemifields.

## General Scientific Summary

This study contributes to further understanding of a significant and prevalent poststroke cognitive impairment. Given the demonstrated link between visuospatial problems and its impact on recovery of other symptoms as well as longer term quality of life outcomes, a better understanding of this heterogeneous disorder and the dissociations between egocentric and allocentric neglect has implications for the development of targeted interventions, particularly for the underresearched allocentric neglect symptoms.

Visuospatial neglect is highly prevalent after stroke. The condition is associated with an attentional deficit where patients fail to orient, perceive, and interact with stimuli on the contralesional side of space. A systematic review of 17 studies comparing the prevalence of neglect acutely, after left brain damage (LBD) and right brain damage (RBD), found that the median prevalence of left-sided neglect (after RBD) was twice as high as for right-sided neglect (43% vs. 21%; Bowen, McKenna, & Tallis, 1999). A recent study on 335 subacute patients (*Mdn* = 28 days poststroke) found incidences of 9% right neglect and 16% left neglect (Ten Brink, Verwer, Biesbroek, Visser-Meily, & Nijboer, 2017), though prevalence reports for right neglect have varied from 2% (Becker & Karnath, 2007) to 65% (Stone, Halligan, & Greenwood, 1993) after LBD. Suchan, Rorden, and Karnath (2012) specifically studied 48 patients with focal LBD and set out to include patients with aphasia in their acute sample. They found a right neglect prevalence of 44% and no statistical difference in the severity of neglect between LBD and RBD patients. Bowen and colleagues (1999) suggested that potential inclusion criteria and selection biases may explain the variability and noted the problematic small samples. In addition, there is a wide range of differing behavioral tests that are currently used to detect neglect (see reviews by Agrell, Dehlin, & Dahlgren, 1997; Halligan, Marshall, & Wade, 1989; Jehkonen et al., 1998; N. V. Marsh & Kersel, 1993). However, there is little consensus about which of these tests are the most sensitive or most appropriate, with some suggesting a combination of tests be required to cover various aspects of detection, dissociations, and severity of neglect (Lindell et al., 2007).

Neglect appears to be a heterogeneous syndrome with dissociable symptoms. One such distinction is between space-based (egocentric) and object-based (allocentric) neglect. Egocentric neglect is where patients fail to attend to the contralesional side of space (with reference to their own body midline). Allocentric neglect is where they fail to attend to the contralesional side of an object in focus. Double dissociations have been reported between patients (Bickerton, Samson, Williamson, & Humphreys, 2011) and even within a single bilateral patient who demonstrated egocentric neglect on one side and allocentric neglect on the other side (Humphreys & Riddoch, 1994). Additionally, egocentric and allocentric neglect have been found to be dissociated in terms of modality. For example, gap detection tasks with circles as targets in both visual and tactile modalities were employed by E. B. Marsh and Hillis (2008). They found four patients displayed egocentric neglect on both visual and tactile tests, and one patient with bilateral lesions exhibited left egocentric visual neglect and right allocentric tactile neglect. There may also be important differences depending on affected side, with work by Kleinman and colleagues (2007) suggesting that, although RBD results mainly in left-sided egocentric neglect, LBD more commonly results in right-sided allocentric neglect. If egocentric and allocentric neglect are dissociated, it is important to identify and utilize tests that can differentially diagnose egocentric and allocentric neglect. Otherwise, arguments that neglect is a unitary syndrome may be due to the lack of tests capable of identifying multiple types of neglect rather than the lack of diversity in neglect impairments themselves. This may help explain why the prevalence of right-sided neglect has been underestimated, because allocentric neglect is often not explicitly assessed (see the overview by Parton, Malhotra, & Husain, 2004). In addition, neuroanatomical lesion studies have supported the dissociation, with findings by Verdon, Schwartz, Lovblad, Hauert, and Vuilleumier (2010), for example, suggesting specific involvement of the right inferior parietal lobule for the egocentric visuospatial component, the right dorsolateral prefrontal cortex for the exploratory component, and deep temporal lobe regions for the allocentric−object-centered component. A recent meta-analysis of 22 lesion-symptom mapping studies of patients concluded that damage to the perisylvian network (pre- and postcentral, supramaginal and superior temporal gyri) and within subcortical structures was associated with egocentric neglect, whereas damage to more posterior lesions including the angular, middle temporal, and middle occipital gyri was associated with allocentric neglect (Chechlacz, Rotshtein, & Humphreys, 2012). We note, however, that all studies here were focused solely on left-sided neglect after unilateral right hemisphere lesions.

**Figure 1 fig1:**
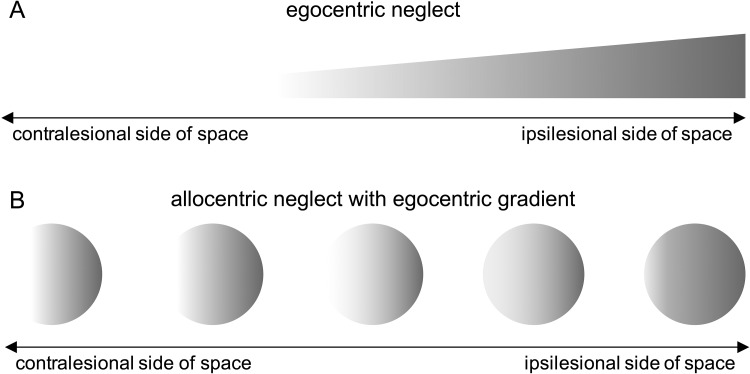
Illustration of how an egocentric spatial gradient (Panel A) could account for allocentric errors (Panel B).

Taking an alternative view, ego- and allocentric neglect may reflect two aspects of a central underlying disorder (Karnath & Rorden, 2012), where allocentric neglect is occurring simply when the attentional window is narrowed to a single object (Driver & Pouget, 2000) or where allocentric biases are modulated by their egocentric position (Karnath, Mandler, & Clavagnier, 2011; Li, Karnath, & Rorden, 2014). Relatively strong correlations between ego- and allocentric neglect have been reported (Rorden et al., 2012), and it has been argued that the two cannot be fully dissociable, given findings where the allocentric deficit was worse for stimuli in the contralesional compared to the ipsilesional side, supporting the notion that allocentric biases occur due to a spatial gradient of attentional weights (see Figure 1).[Fig-anchor fig1]

In this study, we aimed to contrast the prevalence and severity of left- and right-sided egocentric and allocentric neglect in a large sample of acute stroke patients. In addition, we investigated the distributions of allocentric errors in egocentric space to determine whether allocentric neglect can be truly independent from egocentric neglect.

## Method

### Participants and Procedure

This study was designed as a cross-sectional observational study in 419 stroke patients recruited from two acute stroke units at the John Radcliffe Hospital Oxford and the University Hospital Coventry and Warwickshire between 2012 and 2014. Patients completed the Oxford Cognitive Screen (OCS; Demeyere, Riddoch, Slavkova, Bickerton, & Humphreys, 2015) on the acute ward and agreed to be contacted 6 months later for a follow-up visit. Patients were recruited consecutively, depending on practical availability of the patient and researcher at the time. The research team was present for several hours every weekday to recruit the patients and were under clear instructions to try to see everyone who was able to be seen, thereby avoiding a selection bias based on behavioral symptoms or type of stroke. A recent audit of 6 months of our data collection procedures between April and September 2018 (299 total admissions) following the same protocol and criteria demonstrated that our recruitment amounts to approximately 40% of all admissions to the acute stroke unit, with 28% not suitable or too poorly suitable and about 32% missed (not available). Though we collected the clinical admission computerized tomography scans for the sample, only a subset proved to be of high enough quality to determine the more precise lesion location (*N* = 201). We chose not to restrict the analysis to these patients, because the purpose of the study was to assess the prevalence and laterality of ego- and allocentric neglect in an unbiased sample reflecting the clinical reality (see also Massa et al., 2015). This is in contrast with studies investigating the precise relationship between neuropsychological symptoms and lesion location that typically exclude patients with a prior stroke, multifocal lesions or excessive lacunae, minimal lacunar lesions, medical comorbidities, or contraindications for magnetic resonance imaging (e.g., Corbetta et al., 2015; Molenberghs et al., 2009).

**Figure 2 fig2:**
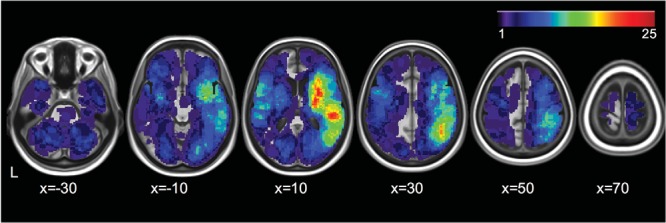
Overlay of the lesions of a subsample where scans were available (*N* = 201 stroke patients) in stereotaxic space (see Varjacic, Mantini, Demeyere, & Gillebert, 2018, for more information on the lesion delineation procedure). The color bar indicates the number of patients with lesions at each voxel. Images are displayed in neurological convention.

Inclusion criteria for the study were that patients should be within 3 weeks of a confirmed diagnosis by the treating stroke physicians to have had an ischemic or hemorrhagic stroke, be able to concentrate for 15 min, and be able to give written informed consent themselves (or witnessed consent in cases of motor problems or agraphia). Of the patients who consented to take part, 366 (87%) provided us with data on the Broken Hearts test of the OCS. Demographics data and stroke information were collected from the medical notes (see Table 1), and a summative overlay of the lesions in the subsample where scans were available is given in Figure 2. Reasons for noncompletion of the Broken Hearts test are given in Table 1 of the online supplemental materials.[Table-anchor tbl1][Fig-anchor fig2]

This study was approved by the National Research Ethics Service (Reference No. 11/WM/0299; Protocol: RP-DG-0610–10/046).

### Measure

The Broken Hearts test is part of the Oxford Cognitive Screen (Demeyere et al., 2015, 2016) and is a highly sensitive cancellation test assessing ego- and allocentric neglect. The task is to strike through the complete shape outlines (*n* = 50) among distractor shapes with gaps on the right (*n* = 50) or the left (*n* = 50) of the contour (see Figure 1 of the online supplemental materials). In the case of hemiplegia, patients use their nonaffected hand to complete the task. The items are positioned semirandomly on an A4 landscape page, equally distributed over a virtual grid. Patients are given up to two practices with demonstration and thorough explanation to ensure they understood the task before starting the test. There is a time limit of 3 min to complete the task.

**Figure 3 fig3:**
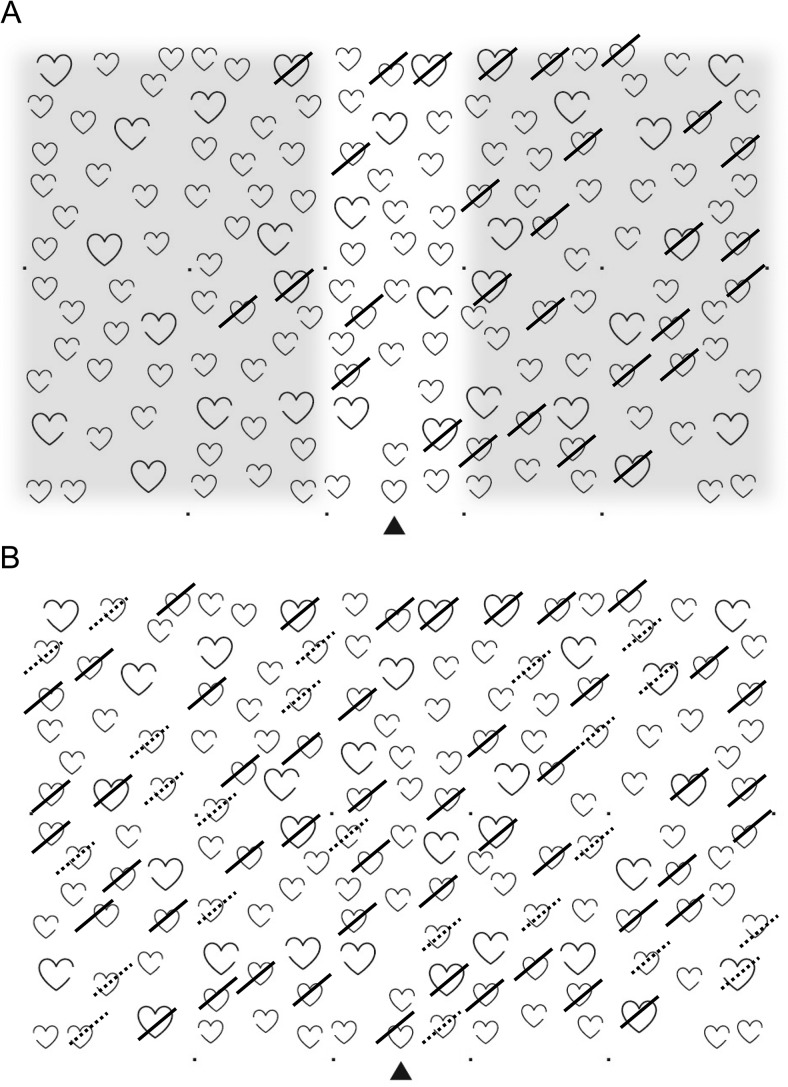
The Broken Hearts test from the Oxford Cognitive Screen (OCS). Panel A: Egocentric neglect is operationalized as an asymmetry value calculated by subtracting the number of hits (full strikes) on the left side of the page from the hits on the right-hand side, also taking into account the total number of hits (the overall correct should be lower than 42, based on the cutoff from normative data). Only the shaded areas are taken into account to calculate the egocentric asymmetry score. Note that the shaded areas have been added only to clarify the scoring; the page presented to the patients contains only the hearts. Panel B: Allocentric neglect is operationalized as an asymmetry score calculated by subtracting the number of false positives (dashed strikes) with a left gap from the number of false positives with a right gap (see also Demeyere et al. 2015).

We calculated the following outcome measures (see Figure 3):

1 *Hits:* The number of correctly canceled full outlines (hearts or apples; maximum 50);
2 *Allocentric errors:* The number of incorrectly canceled distracters (maximum 100).
3 *Egocentric asymmetry:* The difference between the number of hits on the left versus right side of the page. Only the shaded areas in Figure 2A were taken into account. Positive values denote left-sided neglect, and negative values right-sided neglect.
4 *Allocentric asymmetry:* The difference between the number of allocentric errors with a left versus right gap. Positive values denote left-sided neglect, and negative values right-sided neglect.
5 *Egocentric asymmetry of allocentric errors:* The difference between the number of allocentric errors on the left versus right side of the page.

[Fig-anchor fig3]

Cutoffs for impairment were derived from 5th/95th centile scores from the normative data (Demeyere et al., 2015). Patients were considered to have “egocentric neglect” if they had an accuracy score <42 and an absolute egocentric asymmetry score ≥3. Patients were considered to have “allocentric neglect” with an absolute allocentric asymmetry score ≥2.

## Results

### Dissociable Subtypes of Egocentric and Allocentric Neglect in Acute Stroke

**Figure 4 fig4:**
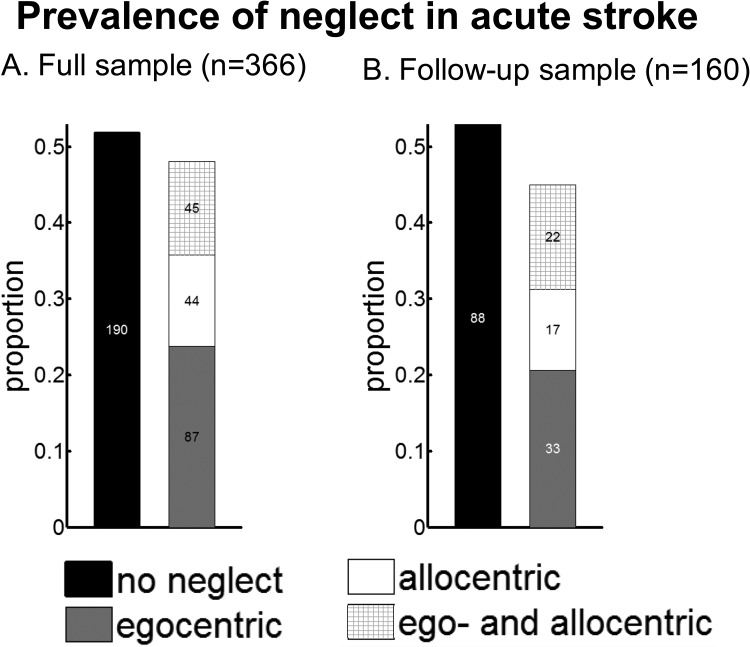
Prevalence of ego- and allocentric neglect in a consecutive sample of 366 acute stroke survivors (Panel A) and in a subsample where follow-up data were collected (Panel B).

Of the patients who completed the Broken Hearts test, 48% demonstrated neglect (see Figure 4A). Half of them presented with only egocentric neglect, one quarter with only allocentric neglect, and a further quarter with both egocentric and allocentric neglect.[Fig-anchor fig4]

**Figure 5 fig5:**
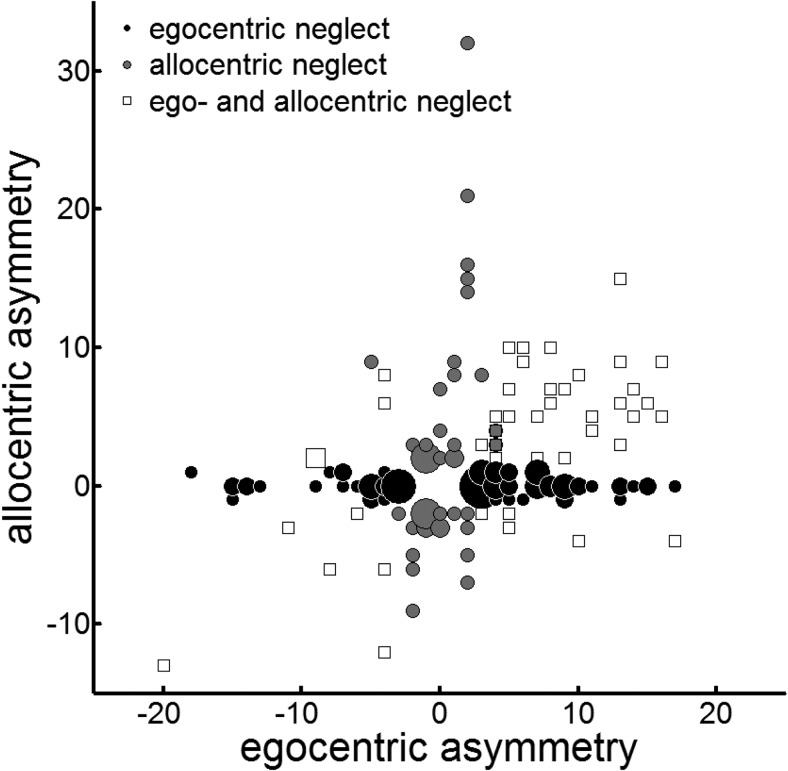
Ego- versus allocentric neglect. Bubble plot of egocentric versus allocentric asymmetry scores in 176 patients with neglect. Positive values reflect left-sided neglect, negative values reflect right-sided neglect. Black circles represent patients with only egocentric neglect (*N* = 87), gray circles represent patients with only allocentric neglect (*N* = 44), and white squares represent patients with both ego- and allocentric neglect (*N* = 45).

To explore the relationship between the severity of ego- and allocentric spatial biases in a more sensitive way, we correlated the ego- and allocentric asymmetry scores in the three groups (see Figure 5). The patients with both ego- and allocentric neglect demonstrated a significant correlation between the asymmetry scores (*N* = 45; *r* = .55, *p* < .0001, large effect size); however, no correlation was present in patients with only egocentric or only allocentric neglect (*N* = 131; *r* = .02, *p* = .78).[Fig-anchor fig5]

**Figure 6 fig6:**
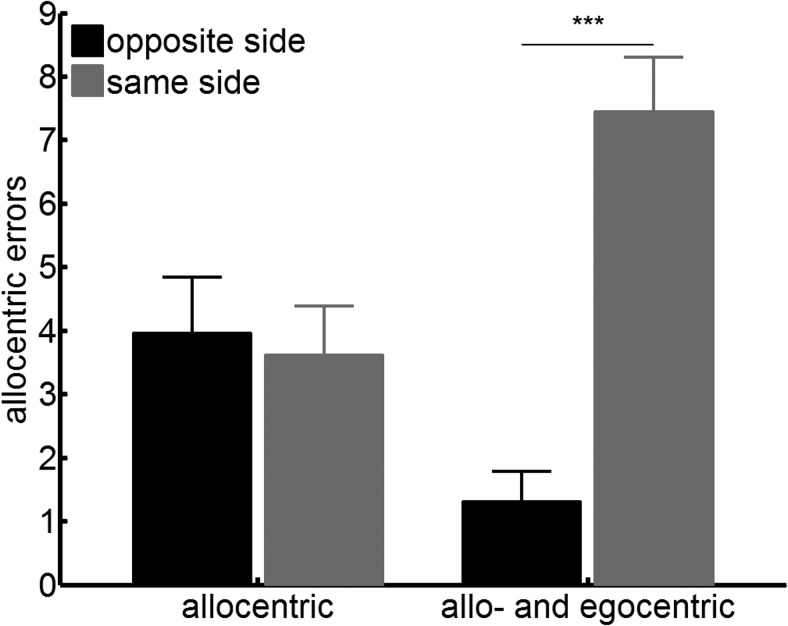
No Egocentric bias in the allocentric errors, demonstrating the opposite pattern predicted in Figure 1. Number of allocentric errors according to the side of space (on the page): numbers of (same vs. opposite side of the allocentric errors) for patients with allocentric neglect only and patients with both ego- and allocentric neglect. Error bars indicate standard error of the mean. *** *p* < .001.

To determine whether there was any egocentric core bias in the allocentric neglect behavior on the test (see the theory proposed in Figure 1), we tested whether the false positive responses made by patients with allocentric neglect were asymmetrically distributed in egocentric space. We examined where on the page the allocentric errors were made. An analysis of variance (ANOVA) with side of space (same vs. opposite side of allocentric errors) and presence of egocentric neglect (allocentric only, ego- and allocentric) showed a main effect of side of space, *F*(1, 76) = 34.91, *p* < .001, η_p_^2^ = .32, and an interaction between the presence of egocentric neglect and side of space, *F*(1, 76) = 43.83, *p* < .001, η_p_^2^ = .37, but no main effect of group, *F*(1, 76) = .34, *p* = .56, η_p_^2^ = .005 (see Figure 6). Post hoc *t* tests suggested that for the allocentric only group, the average number of errors on each side of the page was the same, *t*(42) = .70, *p* = .49, η_p_^2^ = .01. This demonstrates that no egocentric bias was present to explain the allocentric asymmetry, in disagreement with the theoretical framework outlined in Figure 1. In contrast, patients with both ego- and allocentric neglect made fewer errors on the egocentrically neglected side of the page, *t*(34) = −6.82, *p* < .001, η_p_^2^ = .58.[Fig-anchor fig6]

### Prevalence of Neglect

In patients with only egocentric neglect, the prevalence of right-sided neglect (35%) was significantly lower than that of left-sided neglect (65%), χ^2^(1, *N* = 87) = 8.38, *p* < .004. Similar results were obtained in patients with ego- and allocentric neglect: 74% presented with left-sided and 26% presented with right-sided neglect, χ^2^(1, *N* = 45) = 15.11, *p* < .001. However, there was no difference in the prevalence of left-sided (56%) and right-sided (44%) neglect in patients with only allocentric neglect, χ^2^(1, *N* = 44) = .36, *p* = .55.

When considering the co-occurrence of visual field deficits, the Oxford Cognitive Screen (OCS) contains a brief confrontation finger-wiggling task, in each of the four quadrants of the visual field. In our sample, we found 52 patients who showed a visual field deficit on this task. The numbers break down with respect of neglect impairments in the following way: 10 patients did not show any neglect on the Broken Hearts test, whereas 19 presented with egocentric only, seven with allocentric only, and 16 with both ego- and allocentric neglect.

Park et al. (2006) showed that visual defects from an isolated occipital lesion do not cause neglect. These are likely the 10 patients here with an isolated visual field defect. The other 42 patients may well have both a visual field defect and an inattention issue (see also Mort et al., 2003), and in some cases, indeed they may have only very severe inattention, causing them to fail the confrontation task.

Regarding left-sided versus right-sided neglect, a further overview of the co-occurring rates of impairments on the other tasks in the OCS can be found in Table 2 of the online supplemental materials.

For the 160 patients who completed the follow-up assessment 6 months later, the distribution of acute prevalence rates of the different neglect types was proportionally the same as in the full sample of 366 patients (see Figure 4B), demonstrating no selective fallout of patients (reasons for loss of follow-up are given in Table 3 of the online supplemental materials). Fifty-five of these patients demonstrated egocentric neglect at the acute stage, and only 11 were still impaired at follow-up. Allocentric neglect was present in 39 patients acutely and remained present in 10 patients at follow-up. This demonstrates very high recovery rates, at 81% and 74% for egocentric and allocentric neglect, respectively.

### Severity of Left-Sided and Right-Sided Neglect

To directly compare the severity of left- versus right-sided egocentric neglect, we submitted the number of hits and the egocentric asymmetry score to an ANOVA with neglected side (left, right) and the presence of allocentric neglect (egocentric only, egocentric, and allocentric) as variables.

**Figure 7 fig7:**
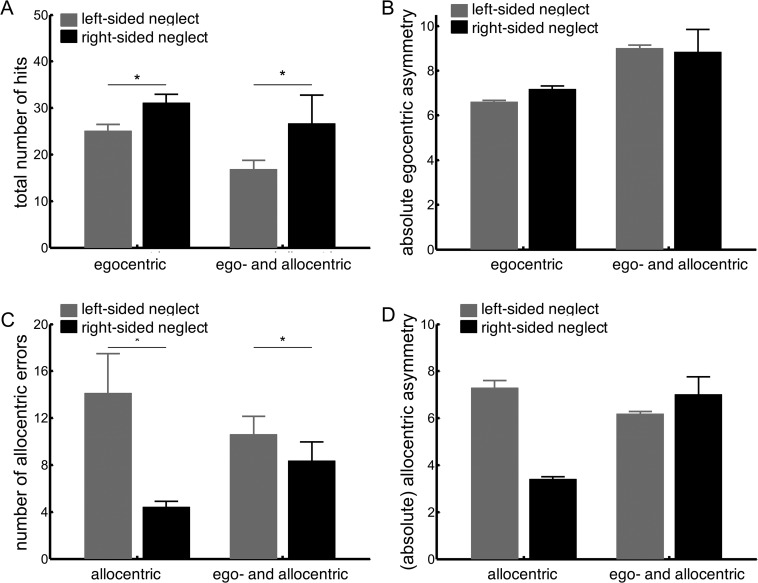
Left- versus right-sided neglect. Severity of egocentric neglect is reflected in the total number of hits (Panel A) and the absolute egocentric asymmetry score (Panel B). Severity of allocentric neglect is reflected in the total number of allocentric errors (Panel C) and the absolute allocentric asymmetry score (Panel D). Error bars indicate standard error of the mean. * *p* < .05.

We observed a main effect of neglected side on the number of hits, *F*(1, 118) = 8.63, *p* = .004, η_p_^2^ = .07, but not on the egocentric asymmetry score, *F*(1, 118) = .04, *p* = .85, η_p_^2^ < .001. Patients with right egocentric neglect demonstrated a higher overall accuracy than did patients with left egocentric neglect, but the strength of the spatial bias did not differ between the groups (see Figure 7, Panels A and B). In addition, we observed a main effect of group: hits, *F*(1, 118) = 5.43, *p* = .02, η_p_^2^ = .04; asymmetry, *F*(1, 118) = 3.76, *p* = .06, η_p_^2^ = .03, showing that patients with ego- and allocentric neglect performed worse on the cancellation task than did patients with only egocentric neglect. The interaction between both factors was not significant: hits, *F*(1, 118) = .48, *p* = .49, η_p_^2^ = .004; asymmetry, *F*(1, 118) = .12, *p* = .73, η_p_^2^ = .001, suggesting that having added allocentric on top of egocentric neglect did not lead to a worse egocentric cancellation performance than did having egocentric neglect only.[Fig-anchor fig7]

To contrast the severity of left- versus right-sided allocentric neglect, we submitted the number of allocentric errors and the allocentric asymmetry score to an ANOVA with neglected side (left, right) and the presence of egocentric neglect (allocentric neglect only, egocentric, allocentric) as variables.

There was a significant main effect of neglected side on the number of allocentric errors, *F*(1, 75) = 3.30, *p* = .039, η_p_^2^ = .06, but not on the allocentric asymmetry score, *F*(1, 75) = 1.39, *p* = .24, η_p_^2^ = .02 (see Figure 7, Panels C and D). The presence of egocentric neglect did not significantly affect the allocentric outcome variables: errors, *F*(1, 75) = .005, *p* = .95, η_p_^2^ < .001; asymmetry, *F*(1, 75) = .91, *p* = .34, η_p_^2^ = .01, and did not significantly interact with the neglected side: errors, *F*(1, 75) = 1.71, *p* = .20, η_p_^2^ = .02; asymmetry, *F*(1, 75) = 3.29, *p* = .07, η_p_^2^ = .04.

To summarize, our data demonstrated a higher prevalence of left compared to right egocentric neglect but a similar prevalence of left compared to right allocentric neglect in this acute sample. The severity of the neglect was more prominent in left-sided neglect (lower accuracy and/or more allocentric errors).

## Discussion

This study set out to address a series of important questions relating to the nature of hemispatial neglect and its prevalence in the early poststroke period. We included a large sample of consecutive acute stroke patients who were not selected based on lesion location or behavioral profile, reflecting the clinical reality of stroke admissions. This is the biggest sample study to assess acute left and right ego- and allocentric neglect prevalence and severity to date.

We found clear evidence to suggest that egocentric and allocentric neglect are subserved by separate underlying processes. First, we documented a double dissociation, with substantive groups of patients with egocentric neglect without allocentric neglect and with only allocentric neglect and no egocentric neglect. In these patients there was no relation between asymmetry values for ego- and allocentric asymmetries. This finding may seem in contrast to a previously reported correlation (*r* = .35) between ego- and allocentric reported by Rorden and colleagues (2012). We note, however, that their sample included only 32 right brain damage (RBD) patients. It may be that this smaller sample accidentally contained more patients with both types of neglect. We indeed also observed a clear correlation in the subgroup presenting with both ego- and allocentric neglect (*r* = .55). It is important to note that in this larger sample, clear dissociable subgroups were present. We further demonstrated that in patients with allocentric neglect only, there was no spatial asymmetry to the distribution of the errors on the page. Where a previous study (Karnath et al., 2011) found an amelioration of allocentric neglect symptoms at more ipsilesional egocentric positions, the three patients in that study presented with both types of neglect. Here, in the allocentric-only patients we did not find any evidence for an egocentric exploration bias. This contradicts the theory proposed by Li and colleagues (2014) that allocentric neglect appears due to an overall spatial gradient (see Figure 1). Instead we suggest that our findings provide strong behavioral evidence for truly dissociable neglect types. The evidence is in line with previous smaller studies (Bickerton et al., 2011; E. B. Marsh & Hillis, 2008; Ota, Fujii, Suzuki, Fukatsu, & Yamadori, 2001) and a striking dissociation in a single case study (Humphreys & Riddoch, 1995). These behavioral dissociations complement the neuroanatomical findings for separable underlying mechanisms (Chechlacz et al., 2010). The previous literature disparities can, in our view, be explained by the undersampling of a heterogeneous disorder. In the present larger sample, the dissociations clearly stand out.

The allocentric neglect observed here may still reflect an object-based neglect or spatial neglect within a smaller reference frame (Driver & Pouget, 2000). Allocentric neglect can indeed be considered in terms of local and global visual representations. For instance, impaired detection of the gap in the Broken Hearts test may stem from impaired attention to one side of the local spatial representation (Humphreys, Gillebert, Chechlacz, & Riddoch, 2013). Arguably, the experience of missing half of an object anywhere in space is existentially different from a global failure to attend to one side of space, and we would hypothesize that allocentric neglect may require a different rehabilitation protocol. Given the separate mechanisms, future research on interventions will need to take this into account. It is possible that traditional interventions aimed at ameliorating egocentric neglect such as cueing and prism therapy may not be effective. More object-based strategies, such as visuomotor feedback, may provide an alternative here (Rossit et al., 2017). We also note the high natural recovery rates found at 6 months follow-up (81% and 74% for egocentric and allocentric neglect, respectively), which further rehabilitation research, should also be taken into account.

In contrast to most studies on neglect, in ours we did not restrict inclusion to patients with only RBD. It is interesting that this unbiased sample in acute stroke revealed high incidences of right-sided ego- and allocentric neglect. Nevertheless, it was clear that left-sided neglect symptoms remained more severe in terms of a lower number of hits and a higher number of allocentric errors.

These findings are in line with long-standing theories of a right hemisphere dominance of hemispatial neglect. Our findings are comparable to those by Stone et al. (1993), Baldassarre et al. (2014), and Ten Brink et al. (2017), who found a considerable number of patients with right-sided neglect in the acute stage, though their symptoms were less severe. Particularly in the chronic stage, the frequency and severity of left-sided neglect was notably higher. This hemispheric difference was initially explained by interhemispheric competition after RBD, leading to an imbalance in attentional control, where the right side of space is overattended (Kinsbourne, 1970). A different account was proposed by Mesulam (1999) in terms of spatial coding for the left side of space being subserved by only the right hemisphere, whereas spatial coding for the right side of space is subserved by both hemispheres. Consequently, left brain damage (LBD) means the right hemisphere can take over the spatial representations. A different account of the right lateralization is that the neglect syndrome encompasses more than a spatial attention bias alone. The presence of impaired sustained attention and working memory in patients with neglect (Husain & Rorden, 2003), processes that are right-lateralized, may contribute to the higher severity and the worse recovery of left-sided as opposed to right-sided neglect (Corbetta & Shulman, 2011). Several studies have now shown the presence of spatial asymmetries in LBD patients without neglect (e.g., see Blini et al., 2016; Gillebert et al., 2011).

In summary, the current study adds to the growing body of evidence viewing neglect as a heterogeneous disorder. There may be a common core spatial deficit, but when it affects egocentric or allocentric space, there are differential and dissociable behavioral profiles.

## Supplementary Material

10.1037/neu0000527.supp

## Figures and Tables

**Table 1 tbl1:** Demographics and Stroke Characteristics of the Study Cohort

Variable	*N*	*M* ± *SD*
Participants	366	
Gender (male/female)	192/174	
Age (years)		73 ± 14
Handedness (left/right/not specified)	28/331/7	
Education level (years)		11 ± 3
Stroke to test interval (days)		6 ± 4
Stroke etiology (ischemia/hemorrhage/not specified)	194/34/138	
Lesion side (left/right/bilateral/not specified)	148/181/24/13	

## References

[c1] Agrell B. M., Dehlin O. I., & Dahlgren C. J. (1997). Neglect in elderly stroke patients: A comparison of five tests. Psychiatry and Clinical Neurosciences, 51, 295–300. 10.1111/j.1440-1819.1997.tb03201.x 9413876

[c2] Baldassarre A., Ramsey L., Hacker C. L., Callejas A., Astafiev S. V., Metcalf N. V., . . . Corbetta M. (2014). Large-scale changes in network interactions as a physiological signature of spatial neglect. Brain: A Journal of Neurology, 137, 3267–3283. 10.1093/brain/awu297 25367028 PMC4240302

[c3] Becker E., & Karnath H.-O. (2007). Incidence of visual extinction after left versus right hemisphere stroke. Stroke, 38, 3172–3174. 10.1161/STROKEAHA.107.489096 17962601

[c4] Bickerton W.-L., Samson D., Williamson J., & Humphreys G. W. (2011). Separating forms of neglect using the Apples Test: Validation and functional prediction in chronic and acute stroke. Neuropsychology, 25, 567–580. 10.1037/a0023501 21574718

[c5] Blini E., Romeo Z., Spironelli C., Pitteri M., Meneghello F., Bonato M., & Zorzi M. (2016). Multi-tasking uncovers right spatial neglect and extinction in chronic left-hemisphere stroke patients. Neuropsychologia, 92, 147–157. 10.1016/j.neuropsychologia.2016.02.028 26948071

[c6] Bowen A., McKenna K., & Tallis R. C. (1999). Reasons for variability in the reported rate of occurrence of unilateral spatial neglect after stroke. Stroke, 30, 1196–1202. 10.1161/01.STR.30.6.1196 10356099

[c8] Chechlacz M., Rotshtein P., Bickerton W.-L., Hansen P. C., Deb S., & Humphreys G. W. (2010). Separating neural correlates of allocentric and egocentric neglect: Distinct cortical sites and common white matter disconnections. Cognitive Neuropsychology, 27, 277–303. 10.1080/02643294.2010.519699 21058077

[c9] Chechlacz M., Rotshtein P., & Humphreys G. W. (2012). Neuroanatomical dissections of unilateral visual neglect symptoms: ALE meta-analysis of lesion-symptom mapping. Frontiers in Human Neuroscience, 6, 230 10.3389/fnhum.2012.00230 22907997 PMC3415822

[c10] Corbetta M., Ramsey L., Callejas A., Baldassarre A., Hacker C. D., Siegel J. S., . . . Shulman G. L. (2015). Common behavioral clusters and subcortical anatomy in stroke. Neuron, 85, 927–941. 10.1016/j.neuron.2015.02.027 25741721 PMC4646844

[c11] Corbetta M., & Shulman G. L. (2011). Spatial neglect and attention networks. Annual Review of Neuroscience, 34, 569–599. 10.1146/annurev-neuro-061010-113731 PMC3790661 21692662

[c12] Demeyere N., Riddoch M. J., Slavkova E. D., Bickerton W.-L., & Humphreys G. W. (2015). The Oxford Cognitive Screen (OCS): Validation of a stroke-specific short cognitive screening tool. Psychological Assessment, 27, 883–894. 10.1037/pas0000082 25730165

[c13] Demeyere N., Riddoch M. J., Slavkova E. D., Jones K., Reckless I., Mathieson P., & Humphreys G. W. (2016). Domain-specific versus generalized cognitive screening in acute stroke. Journal of Neurology, 263, 306–315. 10.1007/s00415-015-7964-4 26588918 PMC4751179

[c14] Driver J., & Pouget A. (2000). Object-centered visual neglect, or relative egocentric neglect? Journal of Cognitive Neuroscience, 12, 542–545. 10.1162/089892900562192 10931777

[c15] Gillebert C. R., Mantini D., Thijs V., Sunaert S., Dupont P., & Vandenberghe R. (2011). Lesion evidence for the critical role of the intraparietal sulcus in spatial attention. Brain: A Journal of Neurology, 134, 1694–1709. 10.1093/brain/awr085 21576110

[c16] Halligan P. W., Marshall J. C., & Wade D. T. (1989, 10 14). Visuospatial neglect: Underlying factors and test sensitivity. Lancet, 334, 908–911. 10.1016/S0140-6736(89)91561-4 2571823

[c17] Humphreys G. W., Gillebert C. R., Chechlacz M., & Riddoch M. J. (2013). Reference frames in visual selection. Annals of the New York Academy of Sciences, 1296, 75–87. 10.1111/nyas.12256 23991639

[c18] Humphreys G. W., & Riddoch M. J. (1994). Attention to within-object and between-object spatial representations: Multiple sites for visual selection. Cognitive Neuropsychology, 11, 207–241. 10.1080/02643299408251974

[c19] Humphreys G., & Riddoch M. (1995). Evidence from unilateral visual neglect. Cognitive Neuropsychology, 12, 283–311. 10.1080/02643299508252000

[c20] Husain M., & Rorden C. (2003). Non-spatially lateralized mechanisms in hemispatial neglect. Nature Reviews Neuroscience, 4, 26–36. 10.1038/nrn1005 12511859

[c21] Jehkonen M., Ahonen J.-P., Dastidar P., Koivisto A.-M., Laippala P., & Vilkki J. (1998). How to detect visual neglect in acute stroke. Lancet, 351, 727–728. 10.1016/S0140-6736(05)78497-X 9504525

[c22] Karnath H.-O., Mandler A., & Clavagnier S. (2011). Object-based neglect varies with egocentric position. Journal of Cognitive Neuroscience, 23, 2983–2993. 10.1162/jocn_a_00005 21391769

[c23] Karnath H.-O., & Rorden C. (2012). The anatomy of spatial neglect. Neuropsychologia, 50, 1010–1017. 10.1016/j.neuropsychologia.2011.06.027 21756924 PMC3348466

[c24] Kinsbourne M. (1970). A model for the mechanism of unilateral neglect of space. Transactions of the American Neurological Association, 95, 143–146. 5514359

[c25] Kleinman J. T., Newhart M., Davis C., Heidler-Gary J., Gottesman R. F., & Hillis A. E. (2007). Right hemispatial neglect: Frequency and characterization following acute left hemisphere stroke. Brain and Cognition, 64, 50–59. 10.1016/j.bandc.2006.10.005 17174459 PMC1949495

[c26] Li D., Karnath H.-O., & Rorden C. (2014). Egocentric representations of space co-exist with allocentric representations: Evidence from spatial neglect. Cortex, 58, 161–169. 10.1016/j.cortex.2014.06.012 25038308 PMC4130897

[c27] Lindell A. B., Jalas M. J., Tenovuo O., Brunila T., Voeten M. J. M., & Hämäläinen H. (2007). Clinical assessment of hemispatial neglect: Evaluation of different measures and dimensions. Clinical Neuropsychologist, 21, 479–497. 10.1080/13854040600630061 17455032

[c28] Marsh E. B., & Hillis A. E. (2008). Dissociation between egocentric and allocentric visuospatial and tactile neglect in acute stroke. Cortex, 44, 1215–1220. 10.1016/j.cortex.2006.02.002 18761135 PMC4297640

[c29] Marsh N. V., & Kersel D. A. (1993). Screening tests for visual neglect following stroke. Neuropsychological Rehabilitation, 3, 245–257. 10.1080/09602019308401439

[c30] Massa M. S., Wang N., Bickerton W. L., Demeyere N., Riddoch M. J., & Humphreys G. W. (2015). On the importance of cognitive profiling: A graphical modelling analysis of domain-specific and domain-general deficits after stroke. Cortex, 71, 190–204. 10.1016/j.cortex.2015.06.006 26232552

[c31] Mesulam M. M. (1999). Spatial attention and neglect: Parietal, frontal and cingulate contributions to the mental representation and attentional targeting of salient extrapersonal events. Philosophical Transactions of the Royal Society of London: Series B, Biological Sciences, 354, 1325–1346. 10.1098/rstb.1999.0482 10466154 PMC1692628

[c32] Molenberghs P., Gillebert C. R., Schoofs H., Dupont P., Peeters R., & Vandenberghe R. (2009). Lesion neuroanatomy of the sustained attention to response task. Neuropsychologia, 47, 2866–2875. 10.1016/j.neuropsychologia.2009.06.012 19545580

[c33] Mort D. J., Malhotra P., Mannan S. K., Rorden C., Pambakian A., Kennard C., & Husain M. (2003). The anatomy of visual neglect. Brain: A Journal of Neurology, 126, 1986–1997. 10.1093/brain/awg200 12821519

[c34] Ota H., Fujii T., Suzuki K., Fukatsu R., & Yamadori A. (2001). Dissociation of body-centered and stimulus-centered representations in unilateral neglect. Neurology, 57, 2064–2069. 10.1212/WNL.57.11.2064 11739827

[c35] Park K. C., Lee B. H., Kim E. J., Shin M. H., Choi K. M., Yoon S. S., . . . Na D. L. (2006). Deafferentation-disconnection neglect induced by posterior cerebral artery infarction. Neurology, 66, 56–61. 10.1212/01.wnl.0000191306.67582.7a 16401846

[c36] Parton A., Malhotra P., & Husain M. (2004). Hemispatial neglect. Journal of Neurology, Neurosurgery & Psychiatry, 75, 13–21. PMC1757480 14707298

[c37] Rorden C., Hjaltason H., Fillmore P., Fridriksson J., Kjartansson O., Magnusdottir S., & Karnath H.-O. (2012). Allocentric neglect strongly associated with egocentric neglect. Neuropsychologia, 50, 1151–1157. 10.1016/j.neuropsychologia.2012.03.031 22608082 PMC3358702

[c38] Rossit S., Benwell C. S. Y., Szymanek L., Learmonth G., McKernan-Ward L., Corrigan E., . . . Harvey M. (2017). Efficacy of home-based visuomotor feedback training in stroke patients with chronic hemispatial neglect. Neuropsychological Rehabilitation. Advance online publication 10.1080/09602011.2016.1273119 28116988

[c39] Stone S. P., Halligan P. W., & Greenwood R. J. (1993). The incidence of neglect phenomena and related disorders in patients with an acute right or left hemisphere stroke. Age and Ageing, 22, 46–52. 10.1093/ageing/22.1.46 8438666

[c40] Suchan J., Rorden C., & Karnath H.-O. (2012). Neglect severity after left and right brain damage. Neuropsychologia, 50, 1136–1141. 10.1016/j.neuropsychologia.2011.12.018 22230231 PMC3348265

[c7] Ten Brink A. F., Verwer J. H., Biesbroek J. M., Visser-Meily J. M. A., & Nijboer T. C. W. (2017). Differences between left- and right-sided neglect revisited: A large cohort study across multiple domains. Journal of Clinical and Experimental Neuropsychology, 39, 707–723. 10.1080/13803395.2016.1262333 27951747

[c41] Varjacic A., Mantini D., Demeyere N., & Gillebert C. R. (2018). Neural signatures of Trail Making Test performance: Evidence from lesion-mapping and neuroimaging studies. Neuropsychologia, 115, 78–87. 10.1016/j.neuropsychologia.2018.03.031 29596856 PMC6018614

[c42] Verdon V., Schwartz S., Lovblad K.-O., Hauert C.-A., & Vuilleumier P. (2010). Neuroanatomy of hemispatial neglect and its functional components: A study using voxel-based lesion-symptom mapping. Brain: A Journal of Neurology, 133, 880–894. 10.1093/brain/awp305 20028714

