# Supplementary material for: Ego- and Allocentric Visuospatial Neglect: Dissociations, Prevalence, and Laterality in Acute Stroke
Source: Neuropsychology. 2019 Mar 21;33(4):490–8. doi: 10.1037/neu0000527 (PMC6487998; doi:10.1037/neu0000527)
Supplement: Supplementary file 1 [file neu0000527_DissociatingNeglect_Suppl_Revision.docx]

Supplementary Table 1: Inclusion on Broken Hearts Test (acute phase of stroke)

| Inclusion | N | % |
| --- | --- | --- |
| Broken Hearts Test completed: | 366 | 87% |
| Broken Hearts Test not completed: |  |  |
| Poor comprehension (receptive aphasia) | 24 | 6% |
| Poor vision | 15 | 4% |
| Task execution not completed (e.g., fatigue) | 11 | 3% |
| Poor eye-hand coordination (optic ataxia) | 3 | <1% |

Supplementary Table 2: Proportion of patients impaired on other OCS subtests in each category: no neglect, any left-sided neglect and any right-sided neglect. (total N= 366)

| OCS subtest | No Neglect | Right-sided neglect | Left-sided neglect |
| --- | --- | --- | --- |
| Picture Naming | 0.25 | 0.42 | 0.37 |
| Semantics | 0.05 | 0.08 | 0.08 |
| Orientation | 0.10 | 0.23 | 0.21 |
| Visual field | 0.04 | 0.14 | 0.32 |
| Sentence Reading | 0.24 | 0.44 | 0.36 |
| Number Writing | 0.28 | 0.53 | 0.42 |
| Calculation | 0.11 | 0.29 | 0.17 |
| Praxis | 0.15 | 0.35 | 0.31 |
| Verbal memory | 0.25 | 0.36 | 0.18 |
| Executive score | 0.13 | 0.18 | 0.25 |

Supplementary Table 3. Reasons for loss to follow up at 6 months post stroke.

|  | N (%) |
| --- | --- |
| Follow up completed | 160 (44%) |
| Follow up not completed: Passed away | 26 (7%) |
| Out of area | 49 (13%) |
| Unable to contact | 46 (12%) |
| Declined | 32 (9%) |
| Information not returned | 21 (6%) |
| Follow up data not available yet | 32 (9%) |

*
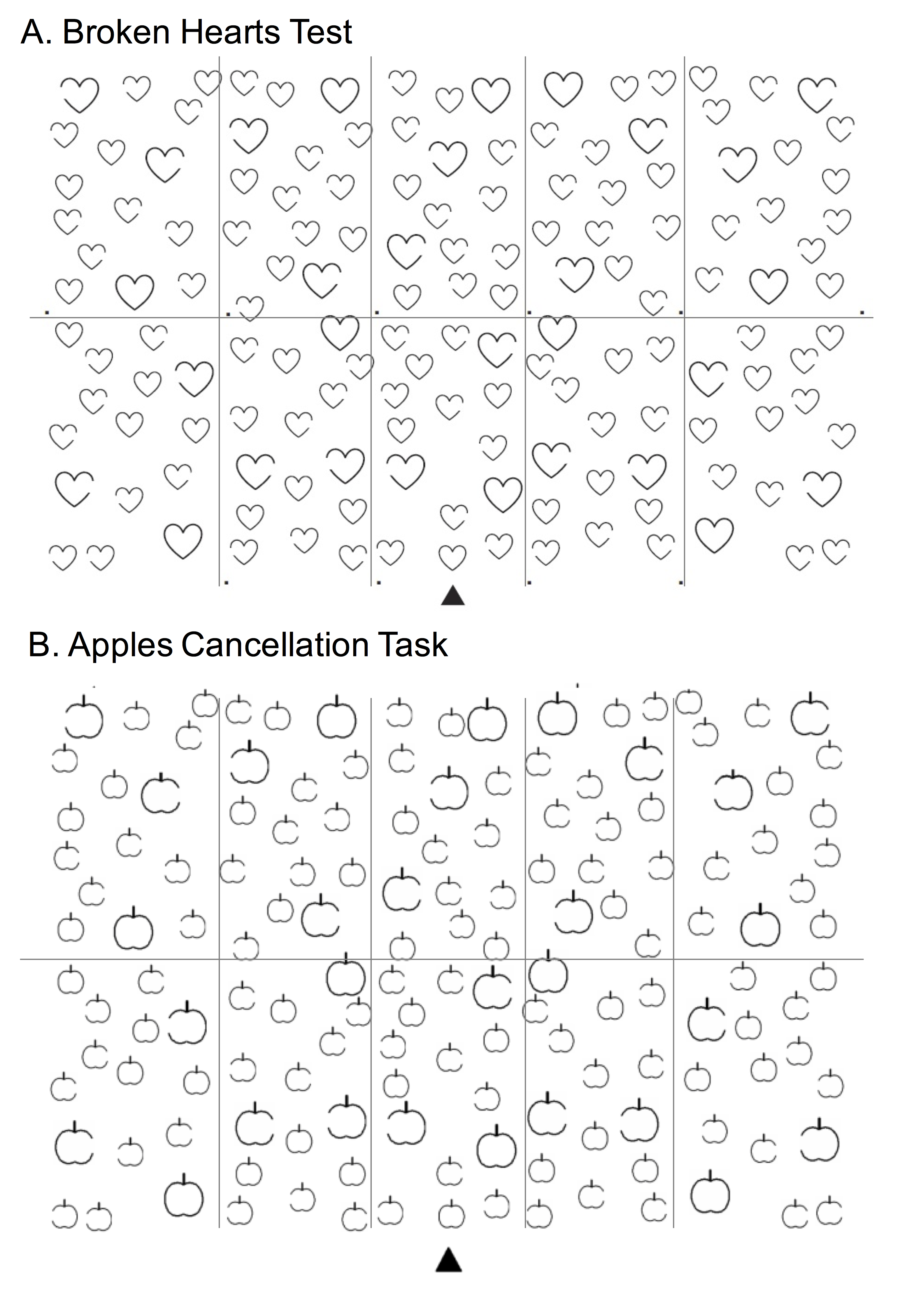
*

**Supplementary Figure 1. Cancellation task**. 150 stimuli (50 targets, 50 distracters with a right-sided gap and 50 distracters with a left-sided gap) are arranged pseudo-randomly on a letter-sized page, in two rows and five columns, with 15 stimuli (5 targets, 5 distracters with a right-sided gap and 5 distracters with a left-sided gap) in each cell. Note that the grid is not visible on the actual test sheet. The Broken Hearts Test is part of the Oxford Cognitive Screen (Demeyere N, Riddoch MJ, Slavkova ED, *et al.* The Oxford Cognitive Screen (OCS): validation of a stroke-specific short cognitive screening tool. *Psychol Assess* 2015;**27**:883–94.).
